# Short-term treatment with nitrate is not sufficient to induce in vivo antithrombotic effects in rats and mice

**DOI:** 10.1007/s00210-016-1308-5

**Published:** 2016-10-14

**Authors:** K. Kramkowski, A. Leszczynska, K. Przyborowski, B. Proniewski, N. Marcinczyk, U. Rykaczewska, D. Jarmoc, E. Chabielska, S. Chlopicki

**Affiliations:** 1 Department of Biopharmacy, Medical University of Bialystok, Mickiewicza 2C Str, 15-222 Bialystok, Poland; 2 Jagiellonian Centre of Experimental Therapeutics (JCET), Jagiellonian University, Bobrzynskiego 14, 30-348 Krakow, Poland; 3 Chair of Pharmacology, Jagiellonian University Medical College, Grzegórzecka 16, 31-531 Krakow, Poland

**Keywords:** Nitrate, Nitrite, Nitric oxide, Thrombosis, Platelet

## Abstract

In humans, short-term supplementation with nitrate is hypotensive and inhibits platelet aggregation via an nitric oxide (NO)-dependent mechanism. In the present work, we analyzed whether short-term treatment with nitrate induces antithrombotic effects in rats and mice. Arterial thrombosis was evoked electrically in a rat model in which renovascular hypertension was induced by partial ligation of the left renal artery. In mice expressing green fluorescent protein, laser-induced thrombosis was analyzed intravitally by using confocal microscope. Sodium nitrate (NaNO_3_) or sodium nitrite (NaNO_2_) was administered orally at a dose of 0.17 mmol/kg, twice per day for 3 days. Short-term nitrate treatment did not modify thrombus formation in either rats or mice, while nitrite administration led to pronounced antithrombotic activity. In hypertensive rats, nitrite treatment resulted in a significant decrease in thrombus weight (0.50 ± 0.08 mg vs. VEH 0.96 ± 0.09 mg; *p* < 0.01). In addition, nitrite inhibited ex vivo platelet aggregation and thromboxane B_2_ (TxB_2_) generation and prolonged prothrombin time. These effects were accompanied by significant increases in blood NOHb concentration and plasma nitrite concentration. In contrast, nitrate did not affect ex vivo platelet aggregation or prothrombin time and led to only slightly elevated nitrite plasma concentration. In mice, nitrate was also ineffective, while nitrite led to decreased platelet accumulation in the area of laser-induced endothelial injury. In conclusion, although nitrite induced profound NO-dependent antithrombotic effects in vivo, conversion of nitrates to nitrite in rats and mice over short-term 3-day treatment was not sufficient to elicit NO-dependent antiplatelet or antithrombotic effects.

## Introduction

For decades, nitrites (NO_2_^−^) and nitrates (NO_3_^−^) were thought to be stable end-products of nitric oxide (NO) produced by NO synthase (NOS) (Bryan 2006). Recently, however, a reductive NO_3_^−^-NO_2_^−^-NO pathway has been proposed as a backup system that acts to maintain NO generation (Kapil 2013).

Compatible with the importance of the reductive pathway for NO production in vivo, even short-term (3 days) supplementation with NaNO_3_ (0.1 mmol/kg daily) was shown to result in a significant increase in plasma NO_2_^−^ concentration in healthy humans and a subsequent fall in diastolic blood pressure (Larsen 2006). Similarly, short-term (3 days) supplementation with dietary nitrate, given in the form of beetroot juice to healthy volunteers, not only reduced systolic and diastolic blood pressure but also improved endothelial function and significantly attenuated ex vivo collagen-induced and ADP-induced platelet aggregation (Webb 2008). Nitrate-induced effects on blood pressure and platelet aggregation were correlated with an increase in plasma nitrite concentration. Furthermore, Kapil et al. (2010) demonstrated that inorganic nitrate given as a single dose (12 or 24 mmol) or in one shot of beetroot juice (containing 5.5 mmol of nitrate) lowered blood pressure. This effect was associated with a dose-dependent elevation in plasma nitrite concentration as well as elevated cyclic guanosine monophosphate (cGMP), confirming that the acute nitrate effect in humans is mediated by the NO-cGMP pathway (Kapil 2010). Obviously, not only short-term but also long-term intake of inorganic NO supplements was shown to have beneficial effects on the cardiovascular system of humans (Zand 2011).

An overwhelming body of evidence suggests that the beneficial effects of nitrate in humans, either upon short-term or long-term treatment (Zand 2011; Mills 2016), involve the enterosalivary nitrate-nitrite-NO pathway. Indeed, it has been reported that ingested nitrate is actively transported from blood to the salivary glands and accumulates in saliva (Tannenbaum 1976; Spiegelhalder 1976; Kortboyer 1994; Qin 2012). Oral bacteria reduces nitrate into nitrite (Tannenbaum 1974; Ishiwata 1975; Duncan 1995; Doel 2005; Kapil 2010), which is swallowed and then absorbed in the intestines, resulting in elevated plasma nitrite concentration (Lundberg 2004; Webb 2008; Kapil 2010; Qin 2012). As reviewed by Lundberg and Weitzberg (2010), nitrate-nitrite conversion in the human oral cavity is metabolized by specific reductase enzymes of the commensal bacteria, whereas nitrite-NO reduction is enhanced by vitamin C, polyphenols, and cytochrome P450 enzymes. Furthermore, a number of mammalian nitrite reductase enzymes have been demonstrated to reduce nitrite to NO, including xanthine oxidoreductase (XOR) (Cantu-Medellin 2013a,b), hemoglobin (Hb) (Huang 2005; Larsen 2011; Kahn 2013), mioglobin (Mb) (Hendgen-Cotta 2014), neuroglobin (Ngb) (Jayaraman 2011), aldehyde oxidase (AO) (Li 2009), sulfite oxidase (SO) (Wang 2010), components of the mitochondrial electron transport chain (ETC), and NOS (Sparacino-Watkins 2014).

Altogether, the evidence suggests that two important pathways exist for NO generation: the first from endogenous L-arginine, involving NO synthase, and the other involving the reduction of nitrate to nitrite that seems to operate particularly in the context of endothelial dysfunction or hypoxia/low pH.

A number of reports have suggested that the beneficial pharmacological effects of nitrate also pertain to rats and mice (Bouaziz-Keteta 2014; Jiang 2014; Carlström 2015; Roberts 2015). However, it remains unclear whether the reductive nitrate-nitrite-NO pathway is as effective in laboratory rodents as it is in humans and whether NO-mediated effects will be seen after short-term treatment, as in humans (Larsen 2006). To explore this question, we comprehensively analyzed the antithrombotic and antiplatelet effects of nitrate as compared to nitrite and analyzed the conversion of nitrate to nitrite in rats after 3-day treatment. We also tested the antithrombotic effects of 3-day treatment with nitrate, as compared with nitrate, in mice.

## Materials and methods

### Animals and the induction of renovascular hypertension

Male Wistar rats were purchased from Charles River Laboratory and housed in the animal house of the Jagiellonian Centre for Experimental Therapeutics (JCET), Jagiellonian University (Krakow, Poland), under a 12-h light/dark cycle. The animals were grouped in cages as appropriate and had access to sterilized tap water and standard rat chow ad libitum. Green fluorescent protein (GFP)-expressing transgenic mice, based on the C57BL/6J strain (GFP mice) (Okabe 1997), were purchased from the Centre for Experimental Medicine in Bialystok. All procedures involving animals were approved by a bioethics committee and conducted in accordance with the institutional guidelines, which are in compliance with national and international laws, including EU Directive 2010/63/EU for animal experiments and the *Guidelines for the Care and the Use of Animals in Biomedical Research* (Giles 1987).

The rats were anesthetized with pentobarbital (40 mg/kg, i.p.). Two-kidney one-clip (2K1C) renovascular hypertension was induced by partial, standardized clipping of the left renal artery (Huang 1981). Six weeks following induction of hypertension, mean blood pressure (MBP) was measured by using the “tail-cuff” method (Panlab, Harvard Apparatus, UE) (Zatz 1990). All rats with elevated blood pressure (SBP/DBP > 140/90 mmHg) were assigned to the hypertensive group. Sham-operated (SO) rats served as the control for 2K1C hypertensive rats. They underwent the same surgical intervention without clipping of the renal artery. Hypertensive rats were divided into groups undergoing experimentally induced arterial thrombosis in vitro experiments.

### Rat model of arterial thrombosis in vivo

The 2K1C rats were anaesthetized with pentobarbital (40 mg/kg, i.p.) and placed in a supine position on a heated (37 °C) operating table. Arterial thrombosis was induced by electrical stimulation of the right common carotid artery, as previously described (Kramkowski 2012). Briefly, the anode, a stainless steel L-shaped wire, was inserted under the artery and connected to a constant current generator. The cathode was attached subcutaneously to the hindlimb. The artery was stimulated (1 mA) for 10 min. Fifty-five minutes after the beginning of stimulation, the segment of the common carotid artery containing the formed thrombus was dissected and opened lengthwise, and the thrombus was completely removed and air-dried at room temperature for 24 h. It was then weighed in a blinded manner.

### Platelet aggregation

Collagen-stimulated platelet aggregation in citrated whole blood was evaluated by using the impedance method, as described previously (Kramkowski 2012), and measured in a whole blood lumi-aggregometer (Chrono-log, USA). Briefly, blood samples were collected from nitrate-treated or nitrite-treated 2K1C rats with electrically stimulated thrombosis. Samples were collected in 3.13 % trisodium citrate at a volume ratio of 10:1. After 15-min incubation at 37 °C with 0.9 % NaCl (volume ratio 1:1), collagen (5 μg/ml) was added. In all aggregation experiments, changes in resistance were registered for 6 min. The extension of the aggregation curve at 6 min was expressed as a percentage of the control response.

### Dynamic thromboxane B_2_ generation

Citrated blood samples from 2K1C rats with electrically stimulated thrombosis were diluted with 0.9 % NaCl at a ratio of 1:1 and stirred in aggregation cuvettes at 37 °C at 1000 rpm. At 0, 20, 40, and 60 min, samples of stirred blood were drawn off and mixed with a cold solution of acetylsalicylic acid (final concentration 500 μM) at a ratio of 1:1. Thromboxane B_2_ (TxB_2_) concentrations in the obtained samples were determined by ELISA, according to the instructions provided by the kit manufacturer.

### Measurement of nitrite, nitrate, and nitrosyl hemoglobin in blood

Plasma concentrations of nitrite and nitrate were measured with the nitrate/nitrite analysis system, ENO-20 (Eicom). ENO-20 employs liquid chromatography with post-column derivatization by using the Griess reagent. Nitrite and nitrate were separated from other substances in matrix on NO-PAK columns (4.6 × 50 mm, Eicom). Nitrate was reduced to nitrite on a cadmium-copper column (NO-RED, Eicom). Nitrite was mixed with the Griess reagent to form a purple azo dye in a reaction coil placed in a column oven at 35 °C, and the absorbance of the dye product was measured at 540 nm. The flow of the mobile phase (Carrier solution, Eicom) was 0.33 ml/min. The Griess reagent was delivered by pump at a rate of 0.11 ml/min. The plasma sample was precipitated with methanol at a ratio of 1:1 (*v*/*v*). Plasma samples were centrifuged at 10,000*×g* for 10 min. Ten microliters of supernatant was injected into the HPLC system.

The concentration of nitrosyl hemoglobin was measured by electron paramagnetic resonance (EPR). EPR spectra of isolated erythrocytes (snap-frozen isolated erythrocytes obtained from whole blood via centrifugation at 1000*×g* and 4 °C for 5 min) were recorded in liquid nitrogen (77 K) by using a Bruker EMX Plus spectrometer operating at X-band, with a 1041HS resonator. The following conditions were used: microwave power = 15.89 mW, time constant = 81.92 ms, modulation frequency = 100 kHz, modulation amplitude = 5*G*, scan time = 20.48 s, scan width = 200*G*, and temperature = 77 K. For each sample, 30 individual scans were averaged. The level of microwave power was determined from the saturation curve of the EPR signal at the centerline of NOHb, which indicated that the signal was not saturated up to approximately to the level of 16 mW. The overlying free radical signals at *G* = 2 were quantified experimentally and deconvoluted to enhance NOHb signal enhance by using an in-house algebraic process. NOHb levels were expressed as EPR amplitude in arbitrary units, normalized to sample weight (259.6 ± 8.8 mg).

### Measurement of coagulation and fibrinolysis parameters in plasma and blood cell count

Prothrombin time (PT), activated partial thromboplastin time (aPTT), and fibrinogen (Fg) levels (Clauss method) were determined according to the kit manufacturer’s instructions by using the Coag-Chrom 3003 apparatus (Bio-ksel, Poland). For fibrin generation, we used a method described previously (He 2001) and modified by us (Buczko 2003). Briefly, fibrin generation curves were created by recalcinating rat plasma samples directly in microplate wells by using CaCl_2_ (36 mM) dissolved in Tris buffer (66 mM Tris, 130 mM NaCl, pH 7.4) at 37 °C. Increases in optical density in the wells (as a result of fibrin generation) were measured with the microplate reader (Biotec EL808, USA) at 1-min intervals for 9 min and expressed as the area under the curve (AUC). Time-point analysis results were calculated as percentages of basal optical density.

Blood cell count was assessed with an Animal Blood Counter (ABC Vet, Horiba, Germany) in blood collected from the left ventricles of 2K1C hypertensive rats after thrombus removal (ex vivo) or untreated 2K1C rats (in vitro experiments).

### Intravital imaging of platelet accumulation after laser injury of endothelium in mice

GFP mice were anesthetized with ketamine and xylazine (i.p.). A midline laparotomy incision was made, and then, the mesentery of the ileum was pulled out of the abdomen and draped over a plastic mound. The mesentery was continuously perfused with 37 °C-warmed phosphate-buffered saline (PBS) to prevent the vessels from drying. Mesenteric venules were identified, and endothelial injury was induced by a 514-nm argon-ion laser (Intelligent Imaging Innovations GmbH, Germany). The laser beam was aimed at the endothelium through the microscope objective lens, and the intensity of laser illumination and duration was kept constant (Falati 2002; Hayashi 2008). Changes in fluorescence intensity were measured automatically by using Slidebook5.0 software. The fluorescence intensity of GFP was normalized to the initial value in each experiment, and then, areas under the curves were calculated for statistics (Intelligent Imaging Innovations GmbH, Germany) (Falati 2002; Hayashi 2008).

### Administration of NaNO_2_ and NaNO_3_

Rats and mice received nitrate or nitrite (0.17 mmol/kg) twice daily for 3 days (by intragastric gavage); the final dose was given 20 min before anesthesia. Sterilized tap water (2 ml/kg) served as a control and vehicle (VEH). The doses of nitrites and nitrates were chosen on the basis of our earlier experiments, which showed that nitrite at a dose of 0.17 mmol is a potent, antithrombotic agent with only weak hemodynamic effects (Kramkowski 2015).

### Chemicals and drugs

The following agents were used: sodium nitrite (NaNO_2_), sodium nitrate (NaNO_3_), calcium chloride (CaCl_2_), sodium chloride (NaCl), and Tris buffer (all Sigma-Aldrich, Germany); pentobarbital (Morbital) from Biovet (Poland); PBS from Biomed (Poland); collagen from Chrono-log (USA); ready-to-use kits for blood cell count from HoribaABX (Germany); oxymetric tests from Ultra Nova Biomedical (USA); routine laboratory reagents for determining PT, aPTT, and Fg levels in rat plasma from HemosIL Instrumentation Laboratory (USA); rat PAI-1 and tissue tPA ELISA kits from Hyphen BioMed (France); and a TxB_2_ ELISA kit from ENZO Life Science. All other products were purchased from Sigma (Gillingham, Dorset, UK).

### Statistical analysis

We used analysis of variance (ANOVA), a multiple comparison test (for normal distribution), and a Bonferroni correction for multiple comparisons after a nonparametric test (for non-normal distribution). A *p* value <0.05 was considered to indicate significance.

## Results

### Effects of nitrate and nitrite on thrombus weight in rat

**Fig. 1:**
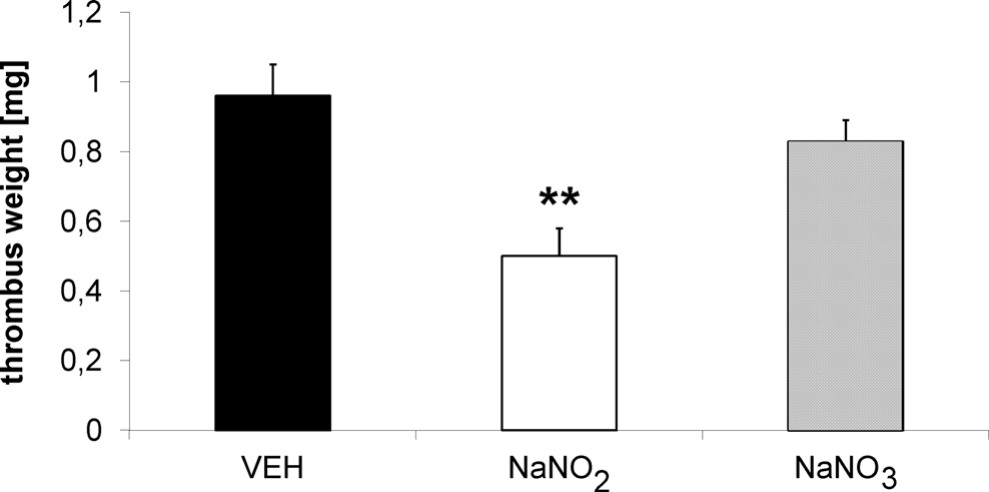
Lack of antithrombotic effect of nitrate compared to nitrite in hypertensive (2K1C) rats after *oral* administration twice daily for 3 days (0.17 mmol/kg); **p < 0.01 vs. VEH

Treatment with nitrate (*n* = 9) at a dose of 0.17 mmol/kg twice daily for 3 days (p.o.) did not affect thrombus weight (TW) (0.83 ± 0.06 mg vs. VEH 0.96 ± 0.09 mg, *n* = 11, ns), while an equimolar dose of nitrite (*n* = 9) decreased TW (TW = 0.50 ± 0.08 mg, *p* < 0.01, vs. VEH) (Fig. 1).

### Effects of nitrate and nitrite on ex vivo platelet function

**Fig. 2:**
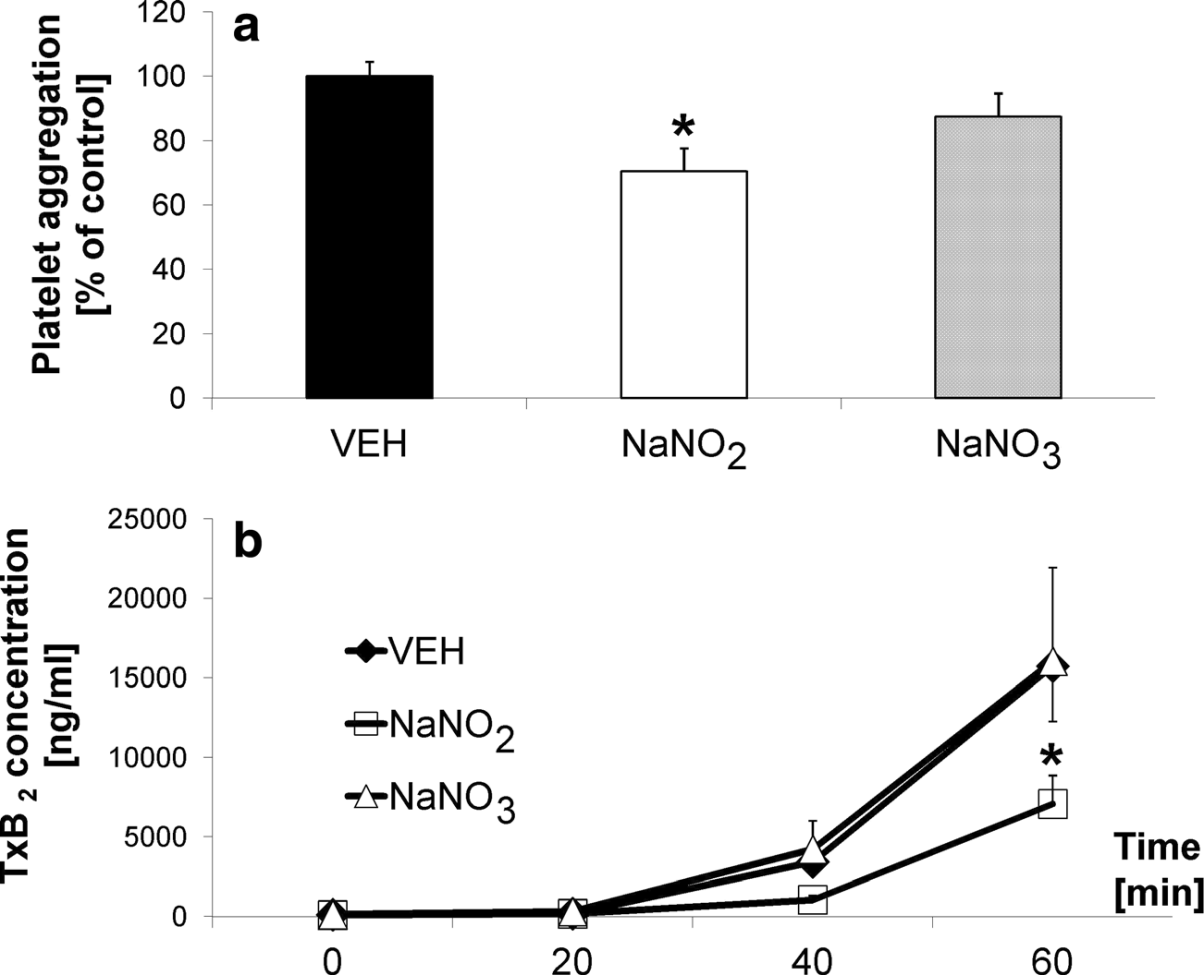
Lack of ex vivo antiplatelet effect of nitrate compared to nitrite: **a** whole blood collagen-stimulated platelet aggregation and **b** dynamic TxB_2_ generation in hypertensive (2K1C) rats after *oral* administration twice daily for 3 days (0.17 mmol/kg); *p < 0.05 vs. VEH

Treatment with nitrate similarly failed to influence collagen-stimulated whole blood ex vivo platelet aggregation (87.50 ± 7.14 % vs. VEH 100 ± 4.46 %, ns), while nitrite treatment inhibited platelet aggregation (70.54 ± 7.14 % vs. VEH, *p* < 0.05) (Fig. 2a). Furthermore, nitrate treatment failed to affect ex vivo dynamic generation of TxB_2_ in full blood assay, while nitrite treatment significantly inhibited generation of TxB_2_ (*p* < 0.05 vs. VEH) (Fig. 2b).

### Effects of nitrate and nitrite treatment on NOHb and nitrate and nitrite concentrations in blood

**Fig. 3:**
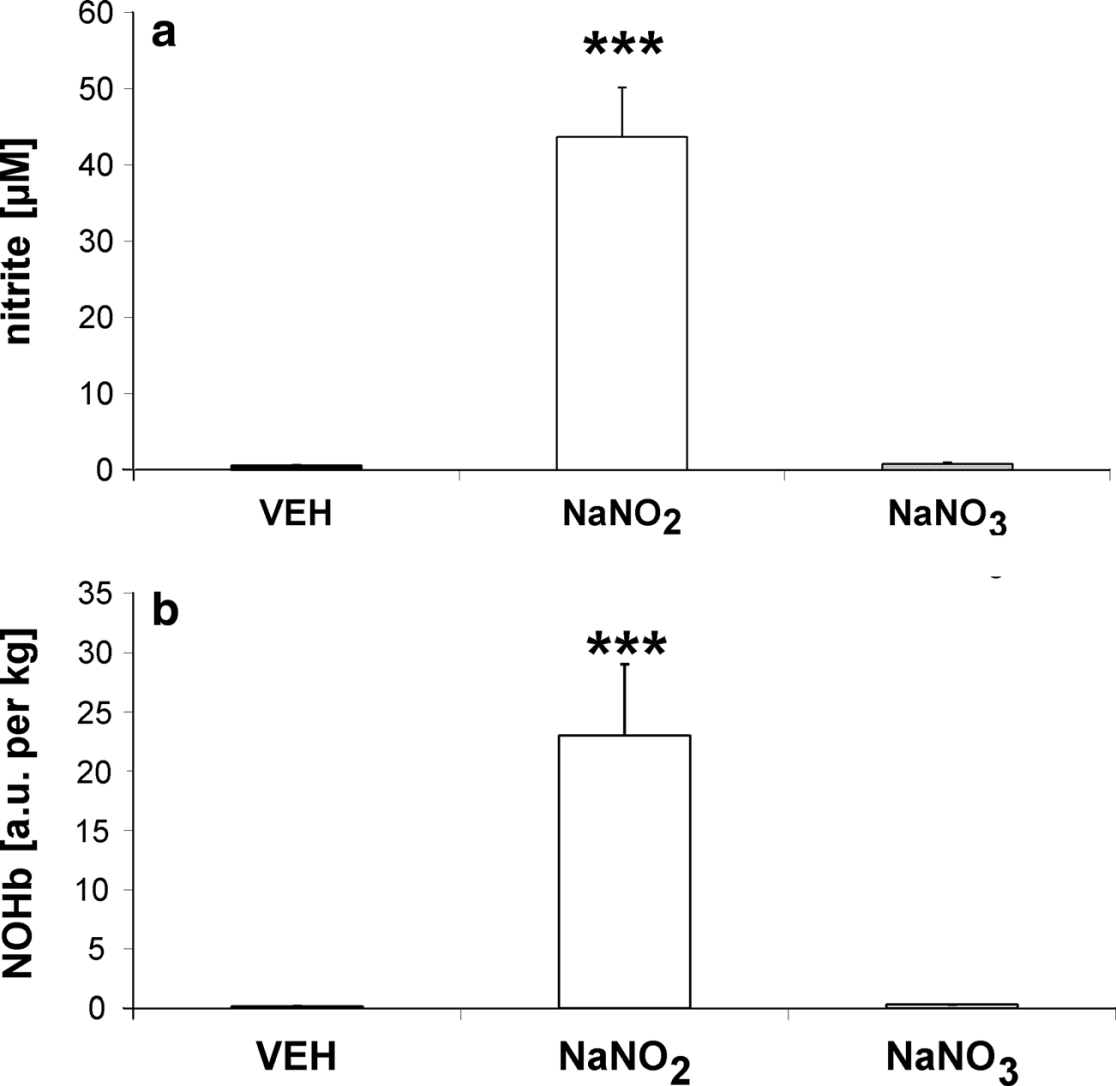
Effect of nitrite and nitrate on **a** nitrite plasma concentration and **b** NOHb content in blood in hypertensive (2K1C) rats (*oral* administration twice daily for 3 days, 0.17 mmol/kg); ***p < 0.001 vs. VEH

As expected, nitrate plasma concentration was profoundly increased in rats treated with nitrate (114.45 ± 6.86 μM vs. VEH 13.70 ± 2.41 μM, *p* < 0.05), while NOHb concentration was only moderately increased (328 ± 6.0 units/mg vs. VEH 231.9 ± 36 units/mg, ns). In the nitrite-treated group (Fig. 3), nitrite plasma concentration was markedly increased (43.71 ± 6.44 μM, *p* < 0.001, vs. VEH 0.61 ± 0.11 μM), as was NOHb concentration (27.100 ± 5514 units/mg, *p* < 0.001, vs. VEH 231.9 ± 36 units/mg).

### Effects on nitrate and nitrite treatment on coagulation and blood cell count

**Fig. 4:**
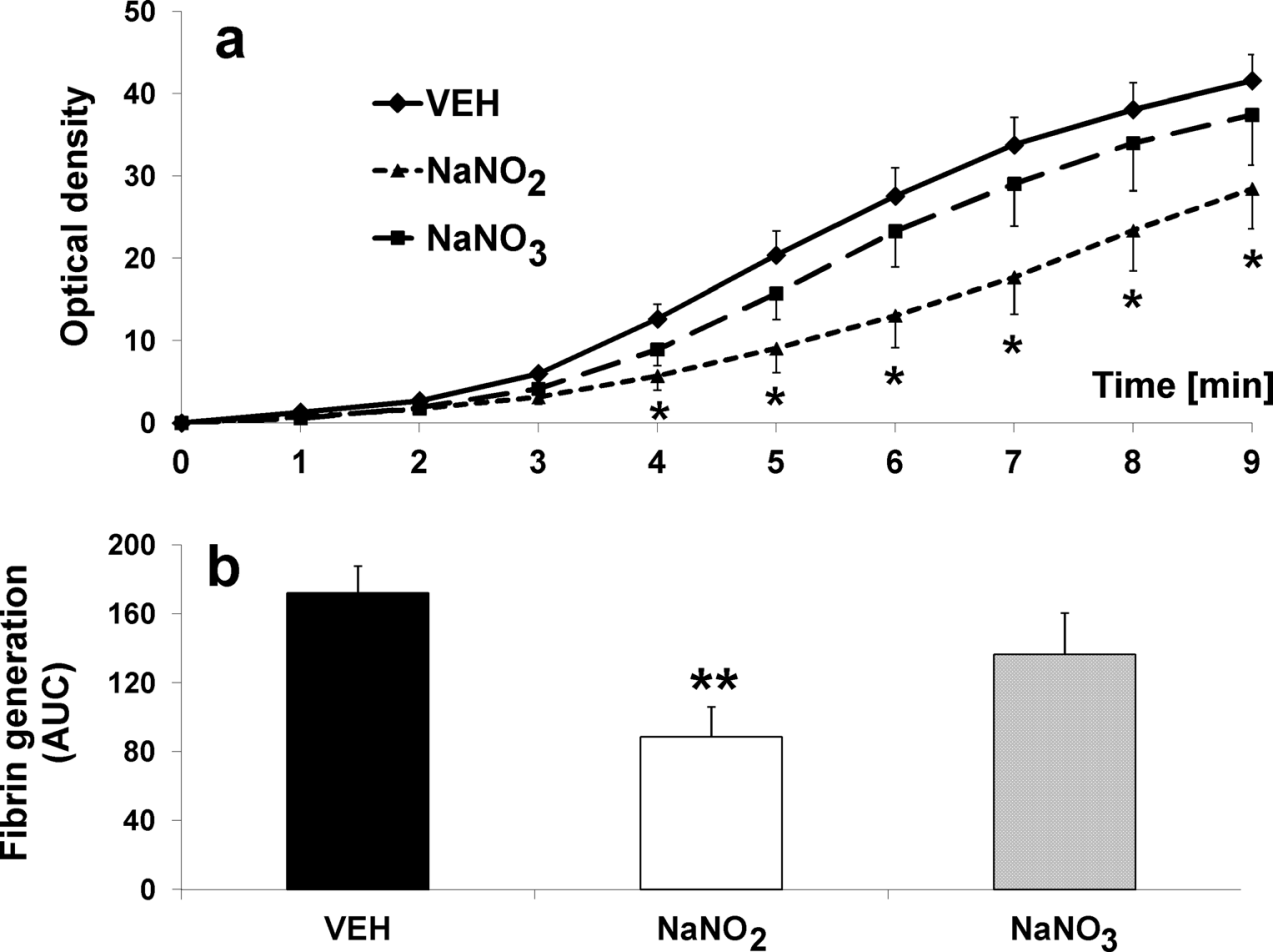
Ex vivo effects of nitrate and nitrite on **a** fibrin generation and **b** area under the curve (AUC) in hypertensive (2K1C) rats (*oral* administration twice daily for 3 days, 0.17 mmol/kg); *p < 0.05, **p < 0.01 vs. VEH

Nitrate treatment did not influence any of the estimated coagulation parameters, while nitrite decreased fibrin generation (*p* < 0.05 and *p* < 0.01 vs. VEH in point-to-point analysis of VEH curve) and decreased area under the curve (88.71 ± 17.44 units^2^ vs. VEH 171.94 ± 15.79 units^2^, *p* < 0.05) (Fig. 4a, b). On the other hand, as shown in Table 1, nitrite treatment slightly prolonged PT but did not change other coagulation parameters such as APTT, TT, fibrinogen level, and QUICK. Neither nitrate nor nitrite influenced blood cell count (Table 2).

**Table 1:** Effects of nitrate and nitrite on clotting parameters in rats

	VEH (2K1C)	3 days NaNO2 0.17 mmol/kg	3 days NaNO3 0.17 mmol/kg
PT [s]	18.1 ± 0.2	19.5 ± 0.6*	18.6 ± 0.4
aPTT [s]	19.1 ± 0.4	18.7 ± 0.6	18.5 ± 0.7
Fg [ng/ml]	2.5 ± 0.1	2.5 ± 0.1	2.4 ± 0.04
QUICK	58.6 ± 2.0	55.0 ± 4.1	57.5 ± 2.6

*PT* prothrombin time, *APTT* activated partial thromboplastin time, *Fg* fibrinogen level, *QUICK* percentage index of prothrombin time (Quick index)

*p < 0.05 vs. VEH

**Table 2:** Effects of nitrate and nitrite on blood cell count in rats

	VEH (2K1C)	NaNO_2_ 3 days 0.17 mmol/kg	NaNO_3_ 3 days 0.17 mmol/kg
WBC [10^6^/μl]	3.33 ± 0.18	2.70 ± 0.19	3.00 ± 0.35
RBC [10^6^/μl]	7.51 ± 0.2	7.10 ± 0.27	7.10 ± 0.10
HGB [g/dl]	13.59 ± 0.35	13.25 ± 0.4	13.23 ± 0.27
HCT [%]	41.58 ± 1.07	40.00 ± 1.52	40.68 ± 1.55
MCV [fl]	55.42 ± 0.31	56.33 ± 0.71	57.5 ± 0.50*
MCH [pg per cell]	18.13 ± 0.15	18.68 ± 0.28	18.68 ± 0.25
MCHC [g/dl]	32.68 ± 0.15	33.17 ± 0.63	32.60 ± 0.45
PLT [10^3^/μl]	623 ± 24	650 ± 31	669 ± 52

*WBC* white blood cells, *RBC* red blood cells, *HGB* hemoglobin, *HCT* hematocrit, *MCV* mean corpuscular volume, *MCH* mean corpuscular hemoglobin, *MCHC* mean corpuscular hemoglobin concentration, *PLT* platelets

### Effects of nitrate and nitrite treatment on in vivo platelet accumulation in mice: intravital imaging

**Fig. 5:**
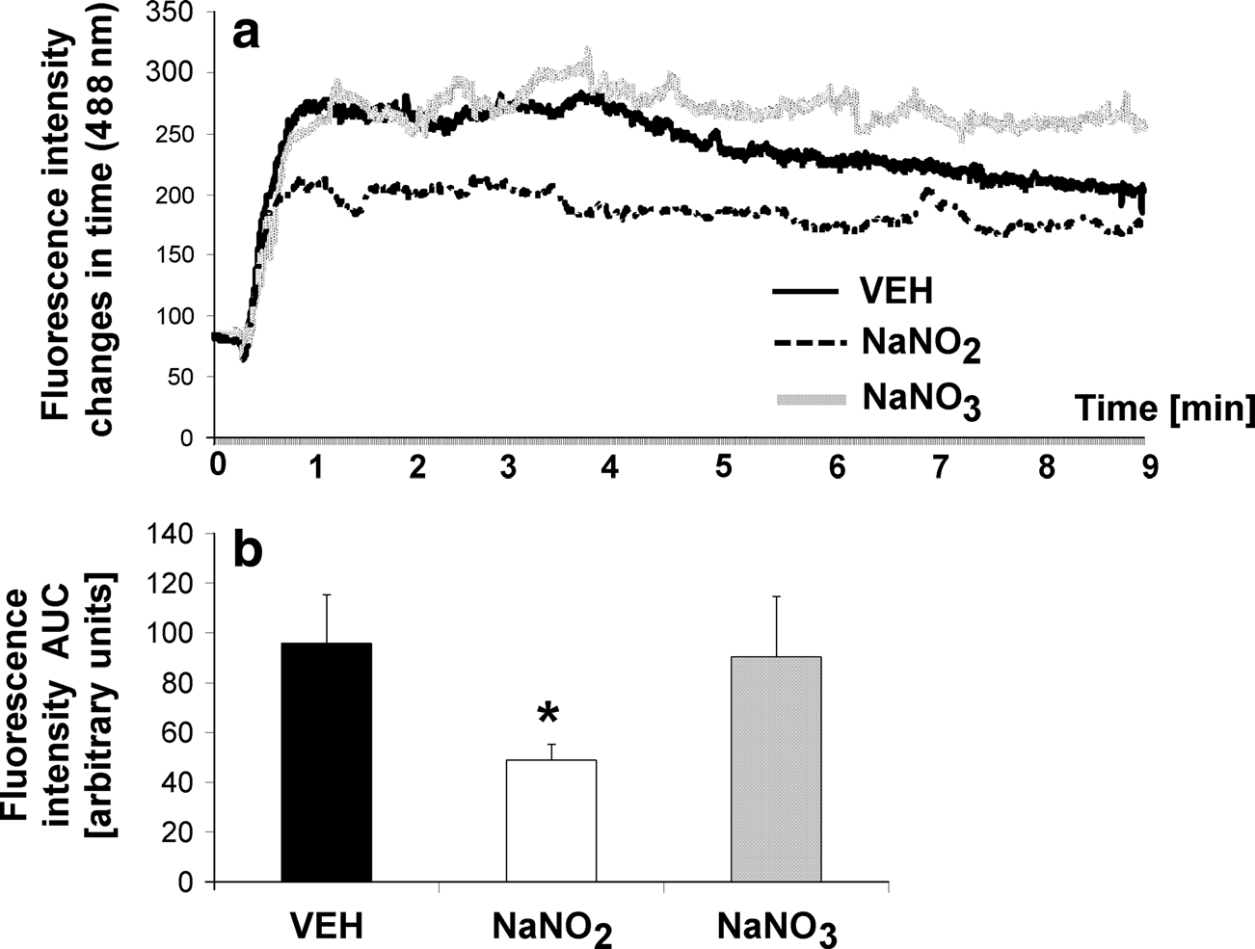
Effects of nitrate and nitrite on **a** kinetics of platelet accumulation in the area of laser-injured endothelium in GFP mice and **b** area under the curve (AUC) (*oral* administration for twice daily for 3 days, 0.17 mmol/kg)

**Fig. 6:**
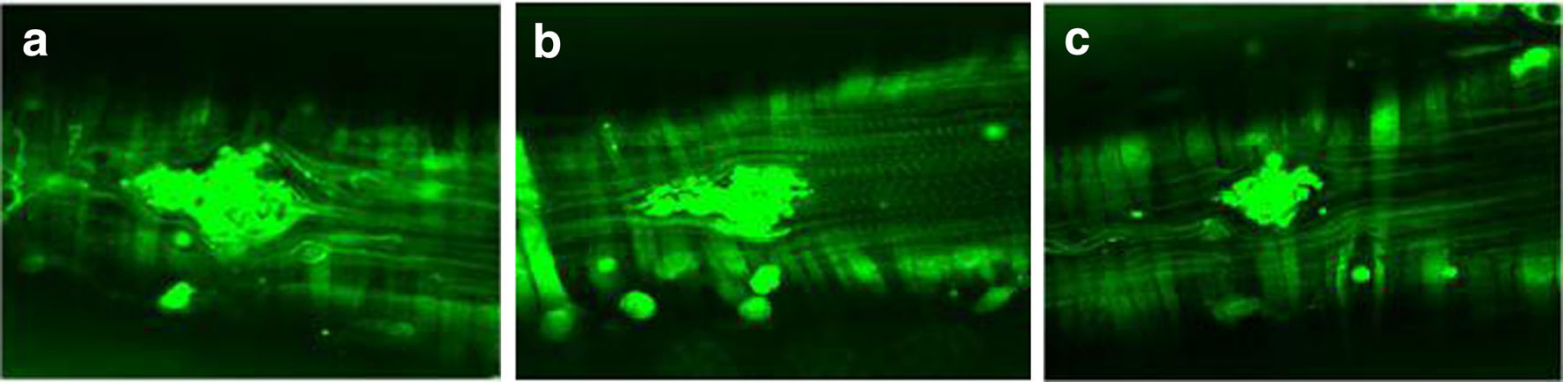
Representative images of thrombi formed 50 s after laser induction in **a** VEH, **b** NaNO_3_ or **c** NaNO_2_ (0.17 mmol/kg per os, 3 days) treated GFP mice

Nitrate treatment did not influence platelet accumulation in the area of the laser-injured endothelium in GFP mice (90.26 ± 24.45 units^2^ vs. VEH 95.76 ± 19.66 units^2^, ns), while nitrite treatment decreased the AUC in the time-course curve of platelet accumulation (49.03 ± 6.31 units^2^ vs. VEH 95.76 ± 19.66 units^2^, *p* < 0.05) (Figs. 5a, b and 6a–c).

## Discussion

In the present work, we demonstrated for the first time that a low dose of nitrate (0.17 mmol/kg, p.o., bid), given as a short-term 3-day treatment, did not display antithrombotic effects in rats and in mice. In contrast, an equimolar dose of nitrite decreased thrombus weight, inhibited ex vivo collagen-stimulated platelet aggregation, inhibited platelet TxB_2_ generation in full blood assay, and inhibited coagulation. Furthermore, we found that nitrite, but not nitrate, decreased fibrin generation. Since the overall coagulation potential depends on active phospholipid surfaces that are exposed by activated platelets, we suggest that nitrite decreases coagulation by an indirect mechanism that is dependent on platelet inhibition, rather than by direct inhibition of clotting factors. Thrombus weight and nitrite plasma concentration correlated with NOHb content in blood, suggesting involvement of NO in the antithrombotic effects of nitrite. Similarly, in mice, nitrate was ineffective, while nitrite decreased platelet accumulation in the area of laser-induced endothelial injury. Although the vasodilatory effect of nitrite has been widely studied, the effect of nitrite on platelet activity has received much less attention so far. Nitrite at a high concentration (500 mM) was reported to inhibit ADP-induced platelet aggregation and to increase cyclic guanosine monophosphate (cGMP) (Laustiola 1991), suggesting that the effects of nitrite on platelets are mediated by NO and the subsequent activation of guanylyl cyclase. The elevation in plasma nitrite levels following the ingestion of a nitrate-rich diet was reported to reduce blood pressure and attenuate the platelet aggregation induced by ADP and collagen in plasma, supporting the importance of nitrite’s effects on platelet activity in humans (Webb 2008). In our experiments in rats, we have clearly demonstrated that nitrite inhibits platelets ex vivo, i.e., inhibits collagen-induced platelet aggregation in whole blood, as well as decreases dynamic generation of TxB_2_. Since arterial thrombosis is strongly platelet-dependent (Kramkowski 2010; Pruolle 2014), our experiments suggest that nitrite’s strong antithrombotic effects are mediated in large part by inhibition of platelets. Indeed, using real-time intravital confocal visualization of thrombosis, we observed highly diminished platelet accumulation after laser injury to the endothelium in mice treated with nitrite. In the same model, nitrate was without effect on platelets.

Altogether, our results suggest that nitrite induces profound NO-dependent antithrombotic effects with short-term treatment in vivo. However, the conversion of nitrate to nitrite in rats and mice was not sufficient to elicit NO-dependent antiplatelet and antithrombotic effects over the short-term period of nitrate treatment shown to be effective in humans (Larsen 2006).

Accordingly, effects of nitrite in rats and mice seem to be similar to those described in humans, including antiplatelet effects mediated by NO (Lasrsen 2006; Webb 2008). On the other hand, we did not observe pharmacologically important antiplatelet or antithrombotic effects of nitrate, underscoring the differences in metabolism between nitrate and nitrite in rats and mice, as compared to humans.

In humans, the half-life of an oral dose of inorganic nitrate is surprisingly long, ranging between 5 and 8 h. On the other hand, nitrate is instantly converted to nitrite, so the increase in plasma nitrite concentration parallels the increase in plasma nitrate concentration (Larsen 2011). It was suggested that the increase in nitrite after nitrate administration is entirely due to enterosalivary circulation of nitrate and nitrate’s reduction to nitrite by commensal bacteria in the mouth (Lundberg 2010). In our previous paper, we have demonstrated that XOR was involved in nitrite reduction into NO in rats (Kramkowski 2015). Here, we did not study the mechanism of nitrite reduction, but we believe that XOR could be involved not only in rats but also in mice, as XOR displays substantially higher activity in rodents as compared to human (Cantu-Medellin 2013a,b). On the other hand, unlike in vitro hypoxic condition (Millar 1998), it is unlikely that XOR contributed to nitrate reduction as the conversion of nitrate to nitrite was minimal either in rats or in mice.

In our study, we administered sodium nitrate or sodium nitrite directly into the stomach, which resulted in elevated plasma nitrate concentrations. As mentioned, in humans, the most important chemical step of this “circulation” is nitrate uptake by the salivary glands, followed by reduction via commensal facultative anaerobic bacteria. In rats, nitrate-reducing bacteria was similarly found on rat tongue, with bacterial density increasing in the direction of the posterior tongue (Li 1997). Interestingly, Hyde et al. (2014) showed that supplementation with nitrate in a high amount (1 g/l) for 7 days in drinking water resulted in a substantial decrease in blood pressure and changes in rat tongue bacterial flora. These changes included a significant increase in nitrate-reducing taxas, for example, *Haemophilus parainfluenzae*, *Granulicatella*, and *Aggregatibacter*. Accordingly, it seems that high nitrate diets may induce changes in the oral microbiome, so as to more efficiently reduce nitrate to nitrite and NO (Hyde 2014). These results are in line with our studies showing that nitrate administered for 10 days at a dose of 0.17 mmol/kg daily decreased thrombus weight to 65 % of control (*p* < 0.05) (Kramkowski 2013). However, as demonstrated in the present work, short-term 3-day supplementation with nitrate (0.17 mmol/kg) was not sufficient to induce NO-dependent pharmacological effects, most likely because the microbacterial flora of the rats and mice used in our experiments could not sufficiently reduce nitrate to nitrite.

It was recently demonstrated that long-term supplementation of drinking water with dietary nitrate reduced stress-induced gastric damage in the bilateral parotid and submandibular gland duct ligature in rats (BPSDL) (Jin 2013). That ligature completely blocked nitrate secretion by the salivary glands, resulting in decreased gastric nitrate (60 %), nitrite (66 %), and NO (62 %) concentrations. After nitrate supplementation in the drinking water for 1 week, the nitrate and nitrite levels in the fasting gastric juice increased. A similar pattern was observed for the level of luminal NO, which was increased by 1000 %. Most importantly, administration of nitrate significantly reduced stress-induced gastric ulcers in BPSDL rats, supporting the notion that the protective effect of nitrate in rats involves enterosalivary circulation. However, nitrate conversion into nitrite through the enterosalivary circulation system seems to require a long period of nitrate exposure to induce the ecological selection of nitrate-consuming bacteria in the gavage. This condition might not be present in the experimental rats or mice fed a standard laboratory animal diet and kept in sterile environment in animal house.

In summary, we have confirmed that nitrite induces profound NO-dependent antithrombotic effects in vivo in mice and rats. However, the conversion of nitrates to nitrite in rats and mice upon short-term 3-day treatment was not sufficient to elicit NO-dependent antiplatelet and antithrombotic effects. This is most likely due to less efficient enterosalivary circulation and sparse microbacterial flora to reduce nitrate to nitrite in rats and mice as opposed to humans, in whom acute NO-dependent effects of nitrate have been demonstrated (Kapil 2013).
